# Improved visible light-driven photocatalytic degradation of methylene blue and methyl red by boron-doped carbon quantum dots

**DOI:** 10.55730/1300-0527.3421

**Published:** 2022-04-01

**Authors:** Bircan HASPULAT TAYMAZ, Çisem KIRBIYIK KURUKAVAK, Handan KAMIŞ, Mahmut KUŞ

**Affiliations:** Department of Chemical Engineering, Konya Technical University, Konya, Turkey

**Keywords:** Carbon quantum dots, hetero-atom doping, photocatalytic degradation, methylene blue, methyl red

## Abstract

The synthesis of fluorescent carbon quantum dots (CQDs) and their applications have attracted great attention due to their excellent properties. Especially, the unique visible-light absorption and photo-induced electron transfer properties make CQDs available in photocatalytic degradation of organic dye pollutants in water resources. Herein, we synthesized nondoped CQDs and boron-doped CQDs (B-CQDs) by hydrothermal method and compared their photocatalytic degradation activity of methylene blue (MB) and methyl red (MR) dyes under visible light irradiation. The characterization outcomes showed that the optical and structural properties can be easily improved by doping with hetero-atom, thereby photocatalytic performance. As expected, the photodegradation performance of both organic dyes in model solutions by B-CQDs was higher than that of CQDs. MB and MR dyes were photodegraded over 95% by B-CQDs in 90 and 120 min visible light irradiation, respectively. Eventually, the results revealed that nondoped CQDs and B-CQDs are excellent candidates for the degradation of organic dyes because of their high photocatalytic performance under visible light illumination.

## 1. Introduction

In recent years, carbonaceous nanomaterials have been gaining significant attention in various application areas. Among them, carbon quantum dots (CQDs), as a new class of carbonaceous nanomaterials, have become a rising member due to their superior properties including, visible-light absorption, water solubility, biocompatibility, low cost, and producibility by easy synthetic approaches [1]. CQDs are photoluminescent nanomaterials having an approximate size in the range of 2–10 nm and their properties could be easily improved by tailoring the size and composition of them [2]. Therefore, the potential of CQDs for applications in optoelectronic devices [3], sensors [4], bioimaging [5], catalysis [6], and so on has emerged. Especially, it has been claimed that the doping of hetero-atoms to CQDs is one of the best routes to enhance electrical and photophysical qualifications of CQDs to enlarge these application areas [7]. Without doping with hetero-atoms, CQDs typically consist of a composition of carbon (C) atoms majority in addition to hydrogen (H) and oxygen (O) atoms [8]. In case of the doping with hetero-atoms, CQDs gain improved physicochemical properties and photoluminescence phenomenon, since the hetero-atoms doped could influence the characteristics of electronic transitions in CQDs due to the extension of orbital overlapping [9].

Up to date, the incorporation of various hetero-atoms (e.g., nitrogen, sulfur, or phosphorus) into CQDs has been studied by hydrothermal/solvothermal method, pyrolysis, solid phase method, microwave-assisted method and so on [10–12]. In comparison to other methods, the hydrothermal method allows low-cost one-step nanomaterial synthesis in addition to its being eco-friendly, nontoxicity, and availability. Several studies have been reported on the synthesis of hetero-atoms doped CQDs by hydrothermal method. For example, Wu et al. reported that nitrogen doped CQDs with high fluorescence stability and quantum yield were synthesized by a one-step hydrothermal method to be used as a fluorescent probe [13]. Taking into account similar studies reported, it could be predicted that using boron as hetero-atom to dope could improve the optical properties of CQDs, similar to nitrogen doped CQDs.

With the increasing industrialization, pollutants discharged from various industries get more attention day by day. Organic dyes, which are nonbiodegradable, have a highly carcinogenic effect on mammals and are toxic to the aquatic medium used in textile [14], paper [15], leather [16], and so on as major pollutants in industry. Therefore, organic dyes must be treated before being discharged into the environment. Many different treatment methods such as adsorption [17], membrane filtration [18], and photocatalysis [19, 20] etc. have been used in dye degradation. In recent years, the photocatalytic organic dye degradation process has attracted much attention. In this method, the photocatalyst induces a light source, an electron (e^−^), and a hole (h^+^) produced on the surface [21]. These e^−^/h^+^ pairs react with very active reactive species and so on; the organic dyes in wastewater are degraded to harmless species [22]. Lately, photocatalysis by CQDs has attracted a great attention to reduce hazardous and toxic compounds in wastewaters due to their excellent photocatalytic performance [23]. Additionally, CQDs offer tunable optical properties, thereby energy band gap value, as well as improved photoelectrochemical properties. To improve their photoluminescence properties, different approaches have been investigated including functionalization, size tuning, hetero-atom doping, or preparation of their nanocomposites. Especially, hetero-atom doped CQDs are considered to be good candidates for expensive traditional semiconductor photocatalysts, as they provide high photoresponsiveness and electron transport properties after doping [24]. Therefore, it is observed that the hetero-atom doped CQDs seem to be promising for enhanced photocatalytic degradation of dyes in an aqueous medium. It is also worth noting that the photocatalytic degradation of organic dyes is economically feasible and environmentally friendly since it requires low energy consumption and low-cost materials.

In literature, the researchers widely focused on synthesizing metal-based photocatalysts for different dye degradation, which could bring additional environmental risk and cost, as well as requiring time-consuming processes. Our work presents a simple fabrication of CQDs using abundant and nontoxic chemicals with excellent dispersibility in aqueous solutions. With the motivation of the above considerations, we present here a comparative study of the degradation of different dyes under visible light irradiation using nondoped (CQDs) and boron-doped CQDs (B-CQDs) synthesized by the hydrothermal method. Even though CQDs have been widely studied due to their unique properties, there are limited reports on photocatalytic degradation of dyes [25, 26].

The aim of this study is to clarify the photocatalytic performance of the B-CQDs for photocatalytic degradation of MB and MR dyes under visible light irradiation. MB and MR dyes were photodegraded over 95% by B-CQDs in 90 and 150 min visible light irradiation, respectively. B-CQDs have excellent photocatalytic performance compared with neat CQDs under visible light irradiation. Furthermore, a possible mechanism is proposed for photocatalytic dye degradation process over B-CQDs after active kinds of scavenge experiments. The improved photocatalytic degradation of dyes studied proves that the incorporation of boron into CQDs enhanced photoresponsiveness and reduced photo-induced e^−^/h^+^ recombination [27]. B-CQDs were obtained from precursors abundantly available and the process efficiency increased. Therefore, economic feasibility was successfully improved. It is worth saying that B-CQDs could be successfully utilized in analytical chemistry and environmental applications.

## 2. Experimental

### 2.1. Synthesis of CQDs and B-CQDs

The hydrothermal method was adopted for the synthesis of CQDs and B-CQDs. Certain amounts of urea (Sigma-Aldrich, 99%) and citric acid (Sigma-Aldrich, 99%) were dissolved in 80 mL distilled water in a beaker for the preparation of CQDs, while a certain amount of boric acid (Merck) as boron source was additionally added into the same solution for the preparation of B-CQDs. After that, hydrothermal synthesis of both CQDs was performed in a stainless-steel cylinder autoclave at 120 °C for 12 h. After allowing to cool to room temperature, the black-green solutions were vacuum evaporated to remove water and unreacted chemicals. Then, CQDs and B-CQDs were dried at 80 °C in an oven overnight.

### 2.2. Characterizations

For the determination of particle size distribution and particle shape, transmission electron microscopy (TEM) images were taken with JEOL JEM-2100 with 200 kV acceleration voltage. To illuminate the changes in optical properties of CQDs and B-CQDs, ultraviolet-visible (UV-Vis) absorption spectrum was recorded at room temperature on a Biochrom Libra S22 UV-vis spectrometer. The photoluminescent (PL) spectrums of CQDs and B-CQDs were recorded using Perkin Elmer LS55.

### 2.3. Determination of photocatalytic activity

The photocatalytic performances of CQDs and B-CQDs were investigated by the degradation of MB (Merck) and MR (Merck) as model organic pollutants. Hundred μL B-CQDs (1 mg/mL) were added to 3 mL model organic pollutants (1 × 10^−5^ M) in a quartz cuvette. The temperature of the mixture was maintained at 25 ± 1 °C. To reach adsorption-desorption equilibrium, B-CQDS-dye mixture was stirred for 60 min in a dark environment. Then, B-CQDs-dye mixture was placed under a visible light source (75 W halogen lamp) to start a photocatalytic reaction. At 15 min intervals, the concentration of dyes was measured as a function of irradiation time using UV-vis absorbance measurements. The photocatalytic performance tests were repeated at least 2 times for CQDs and B-CQDs. The concentration of dyes was estimated with the absorbance intensity decreases of MB and MR at 664 and 523 nm, respectively with a calibration line according to Lambert-Beer law.

## 3. Results and discussion

### 3.1. Characterization

**Figure 1:**
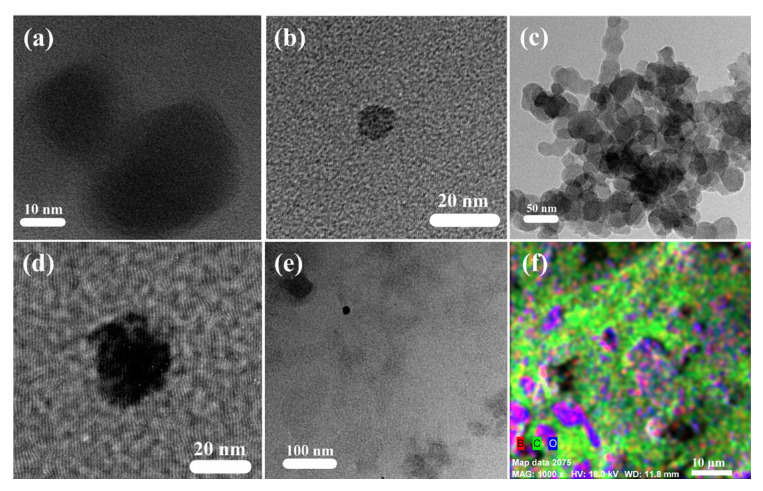
TEM images of the (a and b) CQDs, (c, d and e) B-CQDs and (f) EDX with color mapping of B-CQDs.

The structural analysis of CQDs and B-CQDs synthesized was carried out by the TEM technique. TEM images of CQDs (Figures 1 (a) and (b)) and B-CQDs (Figures 1 (c), (d), and (e)) were given for different scale bars. It could be seen that they were spherical in shape. Their average diameters were calculated to be smaller than 20 nm. The images demonstrated well-dispersed quantum dots. In Figure 1 (d), there are no obvious lattices in B-CQDs as expected, since the CQDs have a mostly amorphous nature, instead of graphitic-CQDs [28]. Undesirably, the cloudy appearance can be seen in Figure 1 (e). This indicates the small agglomeration of B-CQDs. In contrast, other TEM images support the well distribution of both CQDs with no noticeable agglomerates. Additionally, the EDX image with color mapping of B-CQD (Figure 1 (f)) presented that the B and C elements were placed homogeneously over the sample surface, which indicates that the hetero-atoms uniformly dispersed on CQDs.

**Figure 2:**
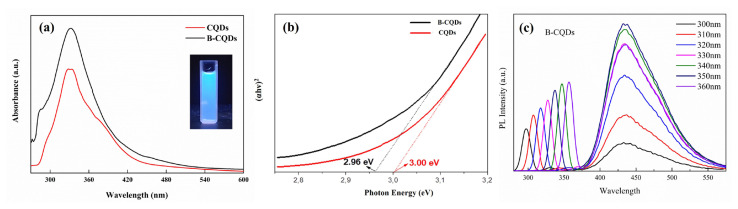
(a) UV-vis adsorption spectra, (b) band gap estimation of CQDs and B-CQD and (c) the PL spectra of B-CQDs with variation of excitation wavelength.

The optical properties were analyzed by recording UV-vis absorption and optical band gap (E_g_) estimation of CQDs and B-CQDs and the results are shown in Figures 2 (a) and (b), respectively. UV-vis absorption spectra of CQDs and B-CQDs showed similar main peaks observed in the UV region. Additionally, the absorption shoulders seen at around 280 nm in both spectra could be attributed to π–π* and n–π*transitions of C=C and C=O bonds, respectively [2]. E_g_ of CQDs and B-CQDs are estimated via Tauc Plots [29, 30] as (αhν)^n^ versus hν from the UV-vis adsorption spectrum where α absorption coefficient, h Planck constant, ν light frequency, and n = 2 for direct band gap material here (Figure 2 (b)). E_g_ values are estimated 3.00 and 2.96 eV for CQDs and B-CQDs, respectively. The interaction between B and CQDs after composite formation ensues in the declined of band gap value. According to the quantum confinement effect, the energy gap values decrease with the increase in the particle size of CQDs, which matches with the findings in TEM characterization results. Further, the decreased bandgap value provides acquiring more light from longer wavelengths [31]. It results to absorb visible light and near-IR range due to narrow band gap and improved performance of CQDs [32].

The PL spectra of B-CQDs with a variation of excitation wavelength (300–360 nm) were given in Figure 2 (c) since the PL intensity is related to the excitation wavelength. The PL spectra showed that the B-CQDs have high PL intensity, which could be related to the availability of sp^2^ sites, aromatic conjugated systems, or structure defects [33]. As well-known, the variation of the emission peak position is dependent on the size of the B-CQDs. Therefore, the PL mechanism can be easily controlled by the size of quantum dots, as well as surface functional groups. As seen, the PL emission spectra showed strong peaks at 430 nm at every excitation wavelength without any shift, which could be related to the uniform size distribution of B-CQDs [34]. In a similar study, the arginine-modified CQDs presented a luminescence peak at around 470 nm with a variation of excitation wavelength (320–560 nm) and it was reported that the particle size distribution was determined to be about 2.5 nm (average diameter) by TEM technique [35].

### 3.2. Photocatalytic activity of B-CQDs

**Figure 3:**
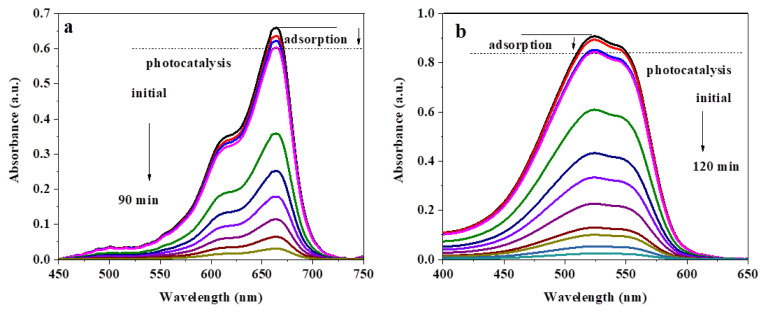
The change of absorption spectra of (a) MB and (b) MR dyes under visible light irradiation using B-CQDs as photocatalyst (dye concentration = 1.0 × 10^−5^ M).

The photocatalytic activity of B-CQDs has identified the degradation of MB and MR dyes under visible light irradiation. Figure 3 indicates the variation of the absorption spectra of a) MB and b) MR dyes under visible light using B-CQDs as a photocatalyst. Before the start of the photocatalytic reaction, B-CQDs −dye suspension was stored in a dark environment, and the results were given as adsorption spectrum in Figure 3. The characteristic absorbance band intensity of dyes suddenly decreases in 15 min after visible light irradiation. The band intensity of MB and MR gradually decreases under visible light after 90 and 120 min, respectively.

**Figure 4:**
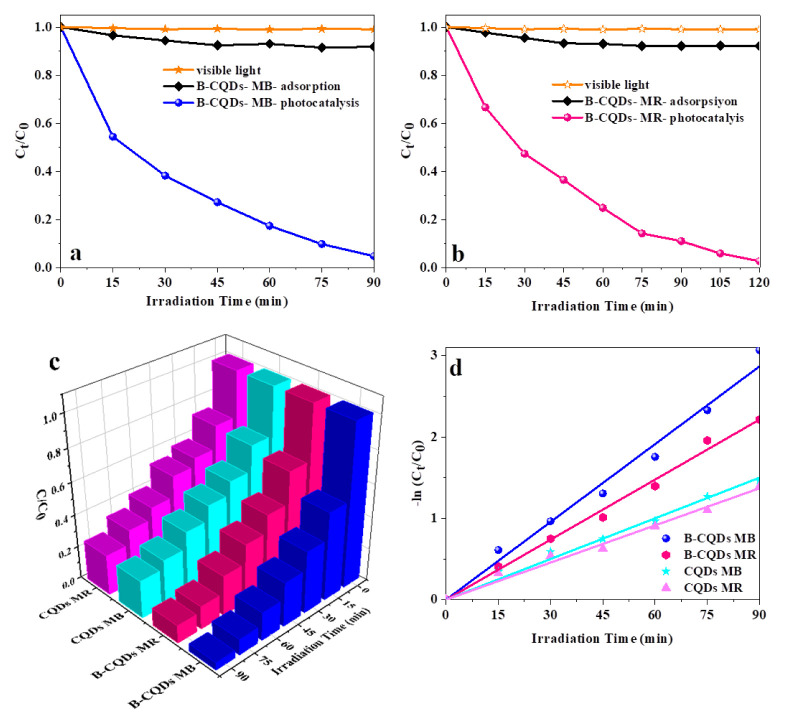
The variation degradation versus irradiation time (a) MB (b) MR under dark and visible light irradiation (c) the comparison of the photocatalytic activity (d) estimation of k_app_ values of CQDs and B-CQDS for degradation MB and MR dyes under visible light irradiation (dye concentration = 1.0 × 10^−5^ M).

Figure 4 (a–b) shows the results of comparative tests to understand the illumination effect on photocatalysis. First, the dyes were stored in a dark environment without photocatalysts, and no change was observed in the absorption spectrum of dyes due to the hydrolysis mechanism. Hydrolysis is not shown in the figure. After dyes are placed under UV and visible light irradiation without photocatalyst to understand the photolysis mechanism, the degradation efficiencies are around 2% for dyes. Before the initiate photocatalytic reaction, B-CQDs-dye suspension is put under the dark and the degradation efficiencies of MB and MR dyes are only 9.3% and 6.8%, respectively (Figure 4). After B-CQDs-dye suspension reaches adsorption-desorption equilibrium, the photocatalytic reaction initiates under visible irradiation. MB dyes were degraded with 95% photodegradation efficiency after 90 min visible light irradiation. Similarly, B-CQDs-MR stored under visible light irradiation within 120 min, a continuous reduction in MR concentration with time was observed with ~97.5% photodegradation efficiency of showing nearly complete degradation. Compared with pure CQDs, B-CODs showed more efficient photocatalytic performance for the degradation of MB and MR dyes, as shown in Figure 4c. The degradation rate constants k_app_ of CQDs and B-CODs for decomposing MB and MR were calculated by the pseudo-first-order kinetics model in Figure 4(d). Table 1 shows the degradation efficiencies and k_app_ values of CQDs and B-CODs for decomposing MB and MR under visible light irradiation.

As shown in Figures 4c–4d and Table 1, B-CQDs show increased photocatalytic activity under visible light irradiation. The k_app_ values of B-CQDs were about two times higher than that of the CQDs for photocatalytic decomposition of MB and MR under visible light irradiation. The increased photocatalytic activity of B-CQDs could be related to improved photogenerated e^−^/h^+^ separation and their effective transfer on the surface, since boron doping increased visible light absorption and quantum yield [36, 37].

**Figure 5:**
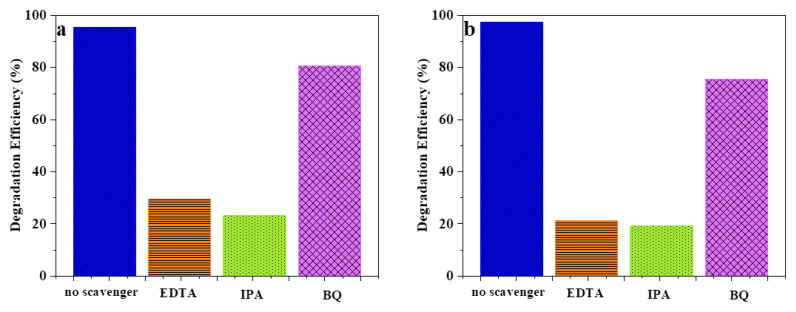
A comparison of photocatalytic activity of B-CQDs in the presence of the various trapping materials (a) MB (90 min) and (b) MR (120 min).

To identify the primary free radicals in the photocatalytic dye degradation process, commonly trapping experiments are used. For this purpose, EDTA, IPA and BQ were added to the photocatalysis reaction medium to trap h^+^, OH^−^ and •O_2_^−^ free radicals. Figure 5 shows the comparison of the degradation efficiency of MB and MR in the presence of different scavengers. The degradation efficiencies of MB and MR are remarkably compressed in the presence of EDTA and IPA. h^+^ and OH^−^ play a more important role in the MB and MR photodegradation process by B-CQDs under visible light irradiation. After adding BQ to the medium, the degradation efficiency of MB and MR has been slightly decreased due to trapping •O_2_^−^ free radicals. In the photodegradation of MB and MR dyes using B-CQDs process under visible light, h^+^ and OH^−^ prominent responsible reactive radicals, while •O_2_^−^ is the secondary free radical.

When boron is introduced to the CQDs, new electron transfer pathways may be formed on the B-CQDs surface [38]. The photogenerated electrons can be grabbed by B-CQDs and their combination reduces remarkably. So, the photocatalytic degradation efficiencies of organic dyes are enhanced in the presence of B-CQDs. A possible photocatalytic degradation of dyes mechanism can be explained below. When the B-CQDs are illuminated under a visible light source, e^−^ are excited and transferred from valance band to conduction band and h^+^ are formed (Eq.1). e^−^ are trapped by oxygen after superoxide radicals (•O_2_^−^) have been generated (Eq. 2). At the same time, photogenerated h^+^ react with water to form hydroxyl radicals (•OH^−^) (Eq. 3). These reactive radicals react with MB and MR dyes and degrade them to carbon dioxide, water, and mineral end products (Eq. 4–5).


(1) $$\text{B-CQDs}+\text{h}\nu \to \text{B-CQDs}({\text{e}}^{-})+\text{B-CQDs}({\text{h}}^{+})$$

(2) O2+B-CQDs (e-)→O.2-+B-CQDs

(3) B-CQDs (h+)+H2O→O.H-+H++B-CQDs

(4) Dye+O.H-→CO2+H2O+mineral end products

(5) Dye+O.2-→CO2+H2O+mineral end products

The photocatalytic performance of the B-CQDs was compared with that of the other reported CQDs based photocatalysts as shown in Table 2. It is observed that the B-CQDs photocatalyst perform enhanced photocatalytic activity compared to the previously reported various photocatalysts for degradation of model dyes under visible light irradiation. Additionally, the excellent photocatalytic activities for the degradation of dyes were faster than previously reported carbon quantum dots (with/without hetero-atom doped) and their composite photocatalysts [41]. With this, especially, B-CQDs demonstrated in this work are good candidates for organic dye degradation and wastewater treatment.

## 4. Conclusion

In summary, CQDs and B-CQDs were synthesized by a facile hydrothermal method, and their photocatalytic performance was studied for the degradation of MB and MR dyes under visible light irradiation. The UV-Vis, PL, TEM, and EDX techniques were used to check the effect of the boron doping on the morphological and optical properties of CQDs synthesized. The particle sizes and the structural properties were examined by TEM and EDX methods. It was revealed that both products were composed spherical in shape with less than 20 nm in diameter. The EDX analysis of B-CQDs presented that there were boron elements confirming the successful synthesis of hetero-atom doped CQDs. Additionally, the findings showed that B and C elements were placed homogeneously over the sample surface. The calculated band gap value from UV-vis spectra of products showed that the boron doping narrowed the band gap value, which leads to absorbing visible light and near-IR range. The PL spectroscopy results revealed that B-CQDs have higher PL intensity and the PL emission spectra of B-CQDs showed strong peaks at 430 nm at every excitation wavelength, which could be related to uniform particle size distribution. Photocatalytic activities of CQDs and B-CQDs were examined by degradation of MB and MR dyes. B-CQDs performed excellent and enhanced photocatalytic performance compared with CQDs under visible light irradiation. MB dye degraded over 95% by B-CQDs in 90 min visible light illumination. Similarly, MR dye nearly completely degraded in the presence of B-CQDs as photocatalyst after 120 min of visible light illumination. At the same time, CQDs were used as a photocatalyst for the MB and MR dyes photodegraded 76% and 62%, respectively in 90 min under visible light irradiation. The incorporation of boron into CQDs enhanced photocatalytic performance due to interaction between boron and CQDs and narrowed bang gap value of B-CQDs. Consequently, facile synthesized B-CQDs could be successfully utilized as a photocatalyst in the photocatalytic degradation of organic dyes under visible light.

## Figures and Tables

**Table 1:** The degradation efficiency %, k_app_ and R^2^ values of CQDs and B-CQDs for the photocatalytic degradation of MB and MR under visible light irradiation (irradiation time fixed up 90 min, dye concentration = 1.0 × 10^−5^ M)

Dye	Photocatalyst	Degradation %	k_app_ (min^−1^)	R^2^
MB	CQDs	76.85	0.0167	0.9874
	B-CQDs	95.32	0.0318	0.9846
MR	CQDs	62.48	0.0118	0.8851
	B-CQDs	89.03	0.0246	0.9923

**Table 2:** Comparison of the photocatalytic activity of various CQDs based photocatalysts.

Photocatalyst	Pollution	Light Source	Degradation (%), time (min)	Ref
CQDs	MB	Visible light	~99.5, 130	[39]
NCQDs	MB	Sunlight	97, 180	[25]
	MG		98, 120	
NCQDs	RhB	Visible light	100, 240	[37]
ZnO/N,S-CQDs	MB	NIR light	72.8, 180	[39]
B-CQDs	MB	Visible light	100, 170	[40]
	RhB		100, 170	
B-CQDs	MB	Visible light	95.32, 90 min	This work
	MR		97.3, 120 min	

RhB: Rhodamine B, NIR: Near-infrared, NCQDs: Nitrogen doped CQDs, ZnO/N,S-CQDs: Zinc oxide/nitrogen, sulphur doped CQDs
